# Does this Need Embolisation? Arterial Bleeding at Anatomically Compressible Sites

**DOI:** 10.1007/s00270-025-04187-4

**Published:** 2025-10-06

**Authors:** Salam Findakly, Kelvin Chang, Natalie Liu, Naradha Lokuhetty, Suhail Wani, Olivia Darby, Warren Clements, Matthew Lukies

**Affiliations:** 1 Department of Radiology, Alfred Health, 55 Commercial Road, Melbourne, VIC 3004 Australia; 2 Central Clinical School, National Trauma Research Institute, Monash University, Melbourne, VIC 3004 Australia; 3 Department of Surgery, Monash University School of Translational Medicine, Melbourne, VIC 3004 Australia; 4 Department of Medical Imaging, Monash Health, Melbourne, VIC 3168 Australia

**Keywords:** Arterial bleeding, Management, Embolisation, Interventional radiology, Trauma, Emergency

## Abstract

**Purpose:**

To assess the efficacy of conservative management for haematomas that demonstrate arterial bleeding, in anatomical regions of the body that are compressible against a firm bony landmark.

**Methods:**

Single-centre retrospective cohort study of patients presenting with arterial bleeding managed conservatively or with either endovascular embolisation or surgical intervention. Patients who were identified to have a CT confirmed haematoma with arterial bleeding, in an anatomical location deemed compressible were included. Conservative management included compression, ice, anticoagulation reversal, and fluid resuscitation. Conservative management was deemed successful if no further intervention was required. Clinical outcomes measured included success of conservative management, length of hospital stay, and 30-day all-cause mortality.

**Results:**

256 patients were included with a mean age of 60 years (SD 21.2). The most common location of bleeding was gluteal (37%) and the most common cause for bleeding was motor vehicle accidents (42%). 67% of cases were managed conservatively as the primary management. Overall, 92.2% of patients were successfully treated with the primary management of choice, with no significant difference between patients managed conservatively or with intervention (92.4% vs. 91.8%, *p* = 0.43). Subgroup analysis of patients presenting in acute shock also demonstrated no significant difference between the conservatively managed group and the group managed with intervention (88% vs. 95% success, *p* = 0.26). The intervention group were more clinically unwell at presentation and had a higher 30-day all-cause mortality (*p* =  < 0.001). There was no significant difference in hospital length of stay.

**Conclusion:**

Conservative management for arterial bleeding in anatomically compressible sites has a high success rate.

**Graphical Abstract:**

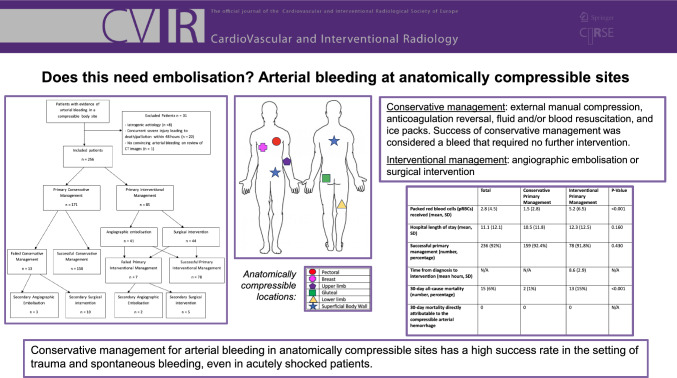

## Introduction

Haematoma is a common diagnosis in the emergency department, often arising in the context of trauma [1]. Presentation can vary from small, superficial haematomas to large or deep soft tissue haematomas precipitating haemodynamic instability and death [2]. Haematomas may also develop spontaneously, particularly with bleeding diathesis, either due to inherited conditions, such as haemophilia, or anticoagulation therapy.

Prompt recognition and localisation of haematomas facilitates effective management, especially with arterial bleeding. Uncontrolled haemorrhage poses significant risks, including haemorrhagic shock, coagulopathy, multi-organ failure, and death [3]. For patients with a high clinical suspicion of arterial bleeding, computed tomography angiography (CTA) is a rapid, non-invasive diagnostic tool [4, 5]. With the advancement of technology in modern medicine, and the accuracy of CTA in detecting even the smallest of arterial bleeding [6], management must not solely rely on imaging findings, but incorporate several factors, including the nature and severity, associated injuries, anatomical location, haematoma size, haemodynamic stability, underlying comorbidities, and medications.

For haematomas in deep or non-compressible sites, embolisation or open surgery with packing and/or vessel ligation are often the only mechanisms to directly arrest the haemorrhage [7]. Conservative management with direct compression and ice can be considered at compressible sites, in addition to reversal of coagulopathy and fluid/blood resuscitation [8–10]. Conservative management leverages the body’s intrinsic haemostatic mechanisms to achieve haemorrhage control, including vasoconstriction, platelet activation and aggregation, and secondary haemostasis via the coagulation cascade, clot retraction, and fibrin stranding [11]. Prior studies have demonstrated positive outcomes with conservative management for arterial bleeding in the retroperitoneum or in small arterial branches of the extremities or the female breast [12–14], however, there is limited data on the efficacy of conservative management including manual compression for arterial bleeding in other anatomically compressible sites.

Our study reviewed cases of arterial bleeding to evaluate whether conservative or invasive approaches—such as angiographic or surgical interventions—were employed, and how these choices influenced patient outcomes, including morbidity, mortality, and hospital length of stay. Additionally, we analysed the impact of various covariates on management decisions and overall outcomes, providing insights into optimizing care. We hypothesise that effective use of extrinsic non-invasive techniques including compression may obviate the need for further interventions.

## Materials and Methods

### Study Design and Setting

This retrospective cohort study was conducted at a Level 1 trauma and tertiary referral centre, with institutional review board approval (733/23), including a waiver of individual patient consent. Patients were identified from the radiology information system (RIS), and individual data collected from electronic medical records (EMR). No funding was provided for this study. The study conformed to the STROBE checklist.

### Patient Selection

**Fig. 1:**
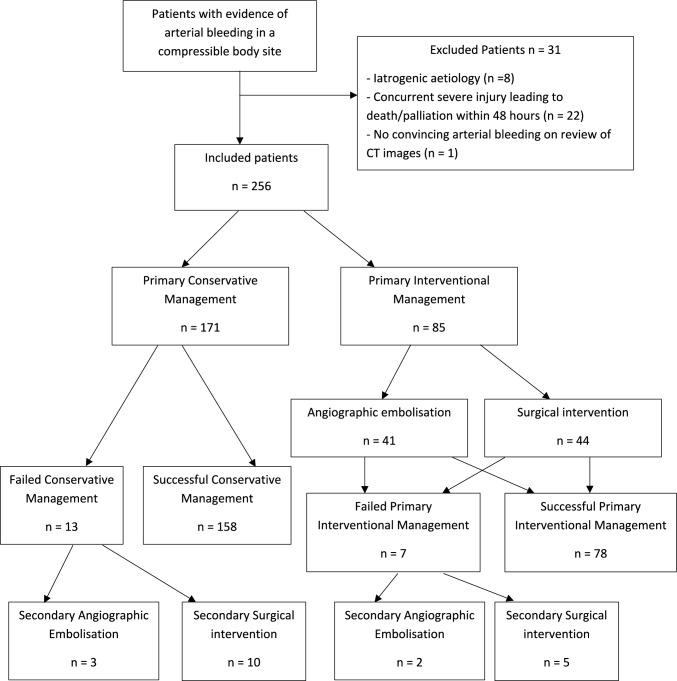
Flow chart showing patient recruitment

The study covered a 14-year period between January 2010 and April 2024 with the following inclusion criteria (Fig. 1).

**Fig. 2:**
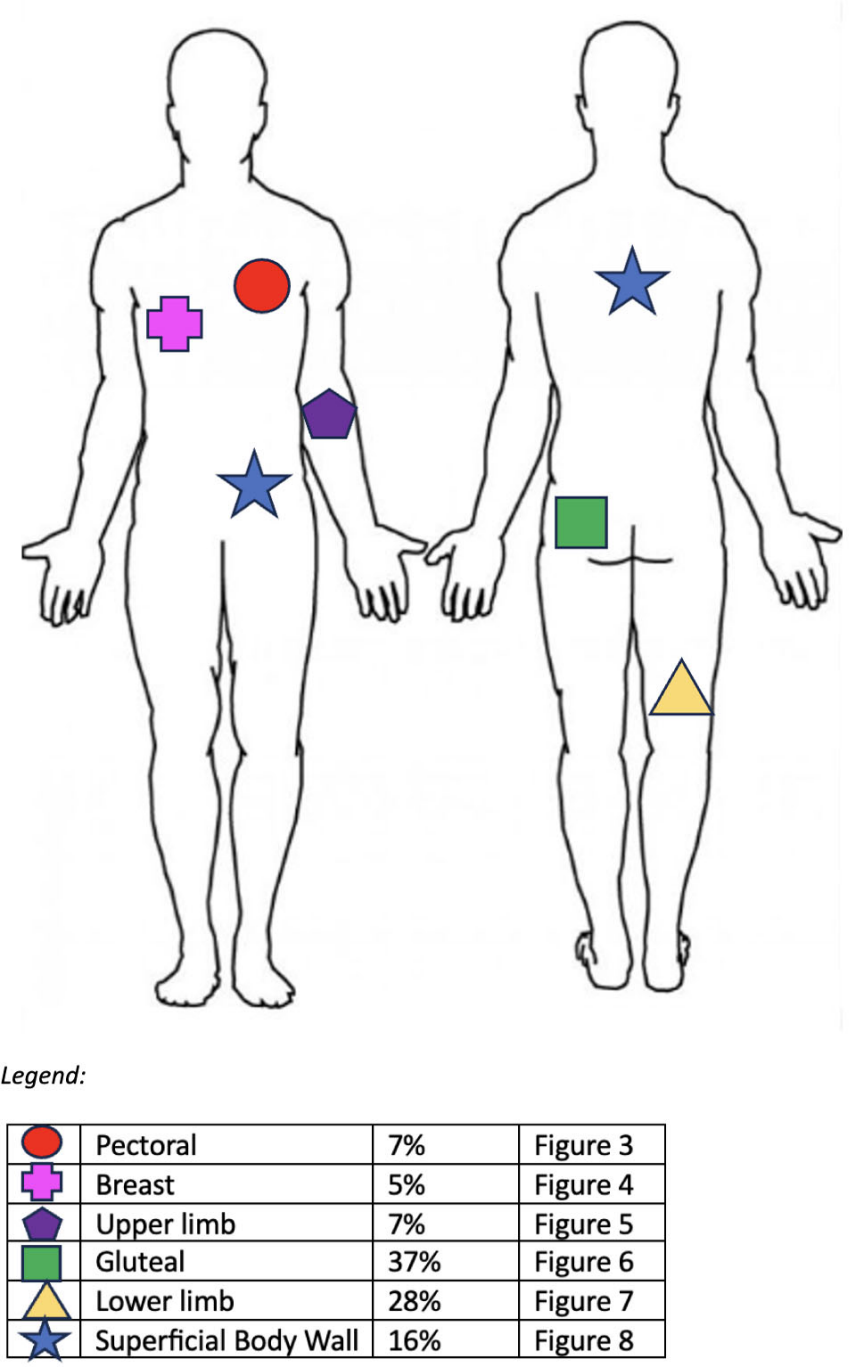
Anatomically compressible locations

- Age ≥ 18 years
- Arterial bleeding on CT imaging, with the report containing the term(s): “active bleeding”, “active arterial bleed”, “contrast extravasation”
- Bleeding in an anatomical location deemed compressible. All included CT images were reviewed by a diagnostic and interventional radiologist to confirm the bleeding was in a compressible anatomical location as defined by our study see below Fig. 2.

Exclusion criteria included:

- Iatrogenic causes of bleeding, such as immediately postoperative or post central line insertion
- Concurrent severe traumatic injuries leading to death/palliation within 48 h, such as non-survivable traumatic brain injuries, as this would make it impossible to assess clinical success
- No convincing arterial bleeding on review of the imaging by a diagnostic and interventional radiologist.

The following data points were recorded based on values at the time of presentation: age, sex at birth, location of bleeding, mechanism of injury, vital signs (lowest systolic blood pressure and highest heart rate), serum markers (haemoglobin, INR, platelets, and renal function), medications (anticoagulation and antiplatelet), primary treatment (conservative, embolisation, surgical), secondary treatment (embolisation, surgical), blood transfusion volume, and outcome (haemoglobin at discharge, hospital length-of-stay and 30 day mortality).

### Definitions

**Fig. 3:**
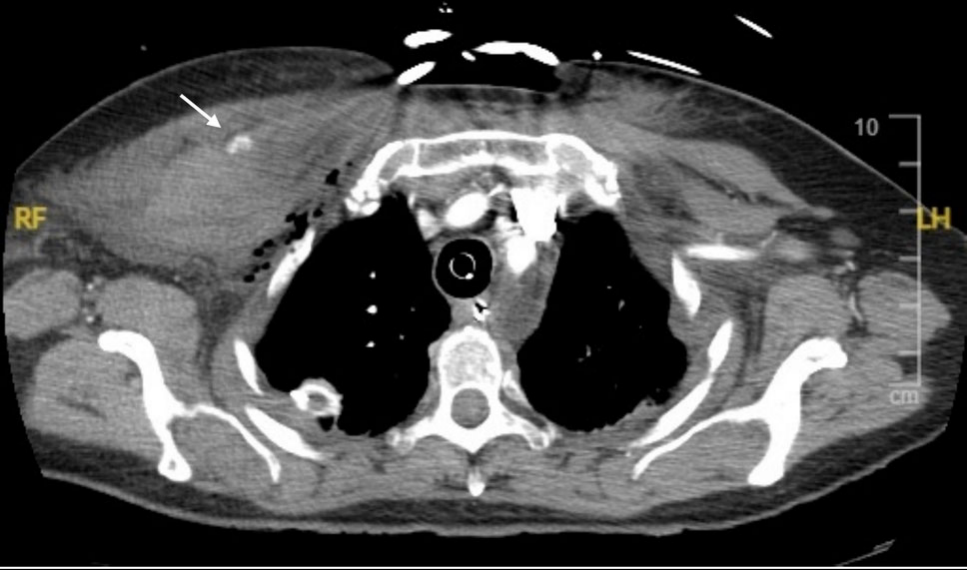
44-year-old male presenting to the emergency department after a motor vehicle accident. Amongst multiple other injuries, the CT scan demonstrated a right pectoral intramuscular haematoma with contrast extravasation (arrow). The patient was treated with endovascular embolisation

**Fig. 4:**
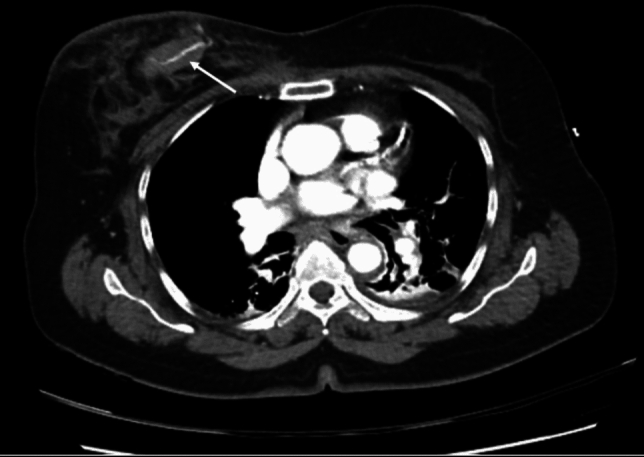
70-year-old female presenting to the emergency department after a motor vehicle accident. The dual-bolus contrast trauma pan scan demonstrated a large right breast haematoma with contrast extravasation (arrow). The patient was successfully managed conservatively

**Fig. 5:**
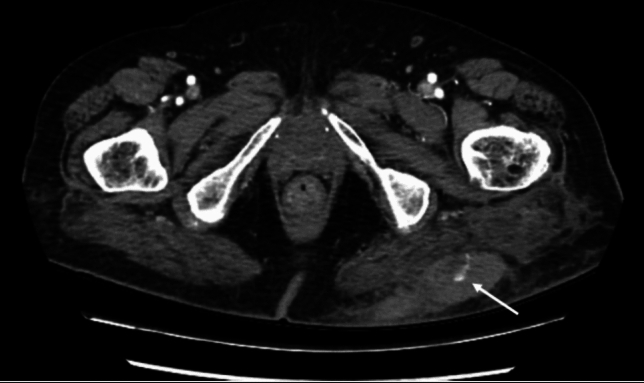
96-year-old female presenting to the emergency department after a fall from standing height. Clinically she had a large left buttock haematoma. Dual bolus CT scan demonstrating a large left gluteal subcutaneous haematoma with contrast extravasation. The patient was successfully managed conservatively

**Fig. 6:**
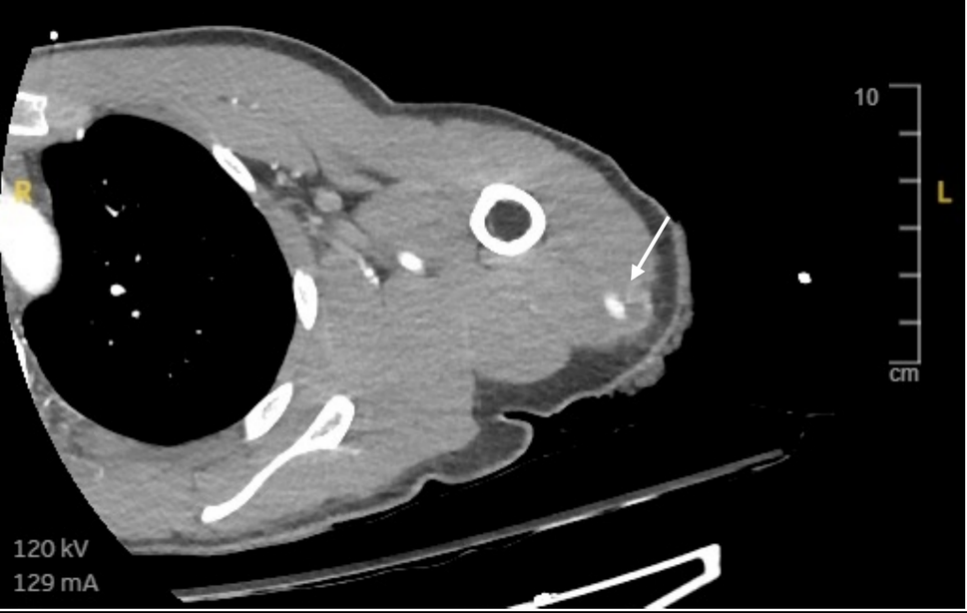
22-year-old male presenting to the emergency department after being stabbed in the left arm. CT scan demonstrated a left upper arm posterior compartment haematoma with contrast extravasation (arrow). The patient was successfully managed conservatively

**Fig. 7:**
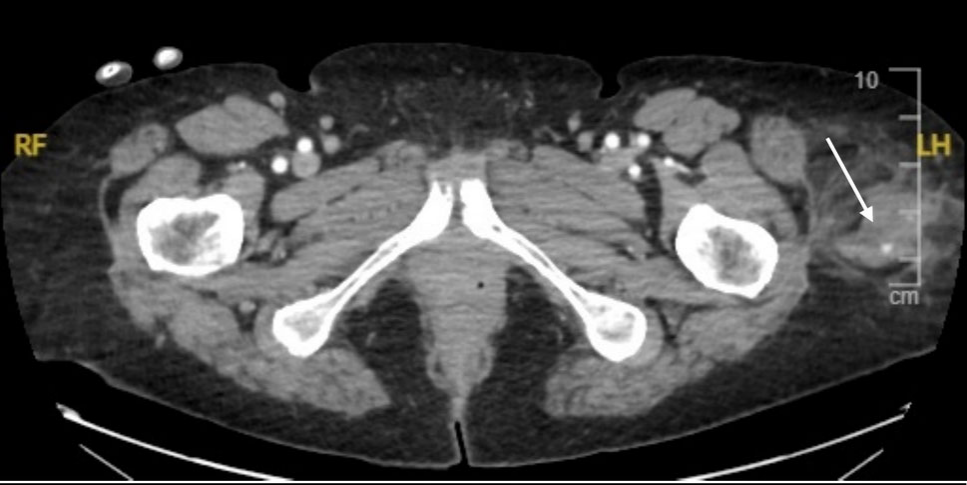
66-year-old female presenting to the emergency department after a fall from standing height. CT scan demonstrated a left lateral hip/upper thigh subcutaneous haematoma with contrast extravasation (arrow). The patient was successfully managed conservatively

**Fig. 8:**
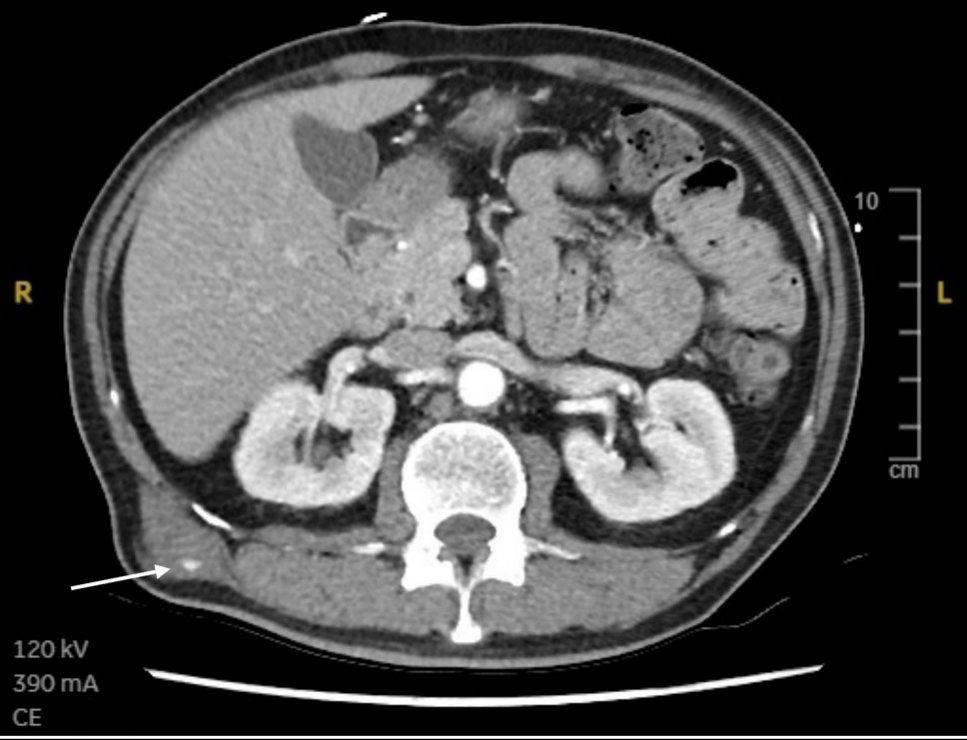
40-year-old male presenting to the emergency department after a motor bike accident. CT scan demonstrated a right latissimus dorsi intramuscular haematoma with contrast extravasation (arrow). The patient was successfully managed conservatively

- *Arterial bleeding:* Any contrast enhanced study demonstrating extravasation interpreted as arterial bleeding (Figs. 3, 4, 5, 6, 7, 8). If > 1 site of arterial bleeding was present in the same patient, management of each site was recorded individually.
- *Compressible site:* Any anatomical location considered to be effectively compressible with manual external pressure against a bony landmark, including the extremities, and superficial axial locations such as the breast, chest wall, back, abdominal wall, and gluteal region (Fig. 2). Abdominal wall locations were only included if they were compressible against bony landmarks such as the iliac crest.
- *Primary management:* Management implemented in the first 24 h.
- *Secondary management:* Any definitive management implemented after the first 24 h that is different to the primary management.
- *Conservative management:* Management of the bleeding site with non-invasive measures such as manual compression, anticoagulation reversal, fluid and/or blood resuscitation, and ice. Successful management was when no further intervention was required. Failure was when secondary management with invasive intervention was required. If conservative management failed within the first 24 h, interventional management was considered as the primary management. If a patient underwent DSA, but no arterial bleed was identified, this was considered interventional management under the principle of ‘intention to treat’.
- *Interventional management*: Angiographic embolisation of the culprit vessel or surgical intervention.
- *Anticoagulation:* Therapeutic doses of either oral or intravenous anticoagulant agents including Warfarin (INR 2–3 or 2.5–3.5 according to indication), low molecular weight heparin (enoxaparin 1 mg/kg BD or 1.5 mg/kg daily), or direct oral anticoagulants (apixaban 5 mg BD, rivaroxaban 20 mg daily, or equivalent).
- *Shock index:* Calculated by dividing heart rate (bpm) by systolic blood pressure (mmHg). A shock index ≥ 1 was considered haemodynamically unstable, an indicator of increased mortality risk [15].

### Outcome Measures

The primary outcome was the rate of clinical success of conservative management. Secondary outcomes included all-cause 30-day mortality, and length of hospital stay.

### Statistics

Data were collated in Microsoft Excel (Microsoft, USA). Statistical analysis was performed using SPSS (version 27.0, SPSS Inc., Chicago, Ill., USA). Data were assessed for normalization and presented as number (percentage), mean (standard deviation) or median (IQR or range). Comparison of means was performed using student’s t-test with *p*-value of less than 0.05 considered significant.

## Results

A total of 315 patients were identified; 230 patients met the inclusion criteria, totalling 256 separate sites of arterial bleeding in anatomically compressible locations (24 patients had two sites of bleeding, 1 patient had three sites, and 1 patient had four sites).

The 256 patients were 67% male (n = 171) and of mean age 60 years (SD 21.2). The most common location of bleeding was the gluteal region (37%) followed by other lower limb regions (28%). The most common cause of bleeding was motor vehicle accidents (42%), followed by fall (32%). The 21% “other trauma” causes mostly comprised of penetrating injuries including stab wounds and gunshot wounds. Amongst the 8 patients with spontaneous bleeds, 3 had haemophilia and 5 were on therapeutic anticoagulation. 23% (n = 60) of patients were on at least 1 anticoagulation medication and 17% (n = 44) on at least one antiplatelet medication (Table 1).

**Table 1:** Demographics

	Total	Conservative Primary Management	Interventional Primary Management	*P*-Value
Patients (number, percentage)	256	171 (67%)	85 (33%)	
Age in years (mean, SD)	60.0 (21.2)	63.3 (20.3)	54.2 (21.8)	< 0.001
*Sex (number, percentage)*				
Male	171 (67%)	112 (65%)	59 (69%)	0.27
Female	85 (33%)	59 (35%)	26 (31%)	
*Aetiology of haemorrhage (number, percentage)*				
Fall	81 (32%)	60 (35%)	20 (24%)	
Motor vehicle accident (MVA)	108 (42%)	72 (42%)	37 (44%)	
Sports injuries	4 (2%)	3 (2%)	1 (1%)	
Other trauma	55 (21%)	32 (19%)	24 (28%)	
Spontaneous	8 (3%)	5 (3%)	3 (4%)	
*Location:* *(number, percentage)*				
Gluteal	95 (37%)	68 (40%)	27 (32%)	
Other lower limb	72 (28%)	45 (26%)	28 (33%)	
Pectoral	18 (7%)	12 (7%)	6 (7%)	
Other upper limb	18 (7%)	6 (3%)	12 (14%)	
Breast	12 (5%)	9 (5%)	3 (4%)	
Superficial chest/abdominal/back wall	41 (16%)	32 (19%)	9 (11%)	
Anticoagulation therapy (number, percentage)	60 (23%)	44 (24%)	16 (21%)	0.27
Antiplatelet therapy (number, percentage)	44 (17%)	33 (19%)	11 (13%)	0.1
Haemoglobin at presentation in g/L (mean, SD)	119 (25.3)	122 (25.3)	115 (24.1)	0.03
Haemoglobin at discharge in g/L (mean, SD)	109 (16.5)	112 (16.3)	105 (16.0)	0.01
Lowest systolic blood pressure (SBP) in mmHg (mean, SD)	103 (22.3)	107 (21.1)	98 (22.3)	< 0.001
Highest heart rate (HR) in beats per minute (mean, SD)	93 (21.7)	89 (20.0)	102 (23.7)	< 0.001
INR (mean, SD)	1.4 (0.6)	1.4 (0.7)	1.4 (0.6)	0.5
Platelet count in g/L (mean, SD)	217 (102.0)	227 (105.9)	197 (92.2)	0.01
Renal function (eGFR) in mL/min/1.73m2 (mean, SD)	74 (18.6)	73 (18.2)	76 (19.4)	0.14
Shock index (mean, SD)	0.96 (0.39)	0.88 (0.35)	1.11 (0.44)	< 0.001
Number of shock index > 1 (number, percentage)	83 (32%)	42 (25%)	41 (48%)	< 0.001

*SD* Standard Deviation. *eGFR* estimated glomerular filtration rate. *INR* International Normalized Ratio. *Shock Index* heart rate (HR) divided by systolic blood pressure (SBP)

67% (n = 171) of cases were managed conservatively as primary management and the remaining 33% (n = 85) were managed with intervention, consisting of embolisation (48%) or surgery (52%). 92.2% of patients (n = 236) were successfully treated with the primary management of choice, including 92.4% of those managed conservatively and 91.8% of those managed with intervention (Table 2).

**Table 2:** Management of patients with arterial bleeding:

	Total	Conservative Primary Management	Interventional Primary Management	*P*-Value
Packed red blood cells (pRBCs) received (mean, SD)	2.8 (4.5)	1.5 (2.8)	5.2 (6.5)	< 0.001
Hospital length of stay (mean days, SD)	11.1 (12.1)	10.5 (11.8)	12.3 (12.5)	0.160
Successful primary management (number, percentage)	236 (92%)	159 (92.4%)	78 (91.8%)	0.430
Time from diagnosis to intervention (mean hours, SD)	N/A	N/A	8.6 (2.9)	N/A
30-day all-cause mortality (number, percentage)	15 (6%)	2 (1%)	13 (15%)	< 0.001
30-day mortality directly attributable to the compressible arterial hemorrhage	0	0	0	N/A

Patients managed conservatively had a higher mean age than those managed with primary intervention (63 vs. 54 *p* =  < 0.001), and on presentation had a higher mean haemoglobin concentration (122 vs. 115 g/L *p* = 0.03), higher mean systolic blood pressure (107 vs. 98 mmHg, *p* =  < 0.001), lower mean heart rate (89 vs. 99 bpm, *p* =  < 0.001), lower shock index (0.88 vs. 1.11, *p* =  < 0.001), and a higher mean platelet count (227 vs. 197 g/L, *p* = 0.01). The conservatively managed group also had a lower number of mean packed RBC transfusions (1.5 vs. 5.2, *p* =  < 0.001) and lower 30-day all-cause mortality (2% vs. 13%, *p* =  < 0.001). Length of hospital stay for the conservatively managed group was shorter, however this was not significant (*p* = 0.16). Both groups had a similar mean INR (1.4) and there was no significant difference in the proportion of those taking anticoagulation (*p* = 0.270) and/or antiplatelet therapies (*p* = 0.100).

Overall, primary management failed in 20 patients, necessitating secondary intervention. 13 (8%) of these were initially managed conservatively and 7 (8%) with intervention (*p* = 0.430). Of the 13 initially managed conservatively, 3 underwent secondary embolisation and 10 underwent secondary surgical intervention.

30-day all-cause mortality was 6% (15 patients), most commonly due to haemorrhagic shock and sepsis. 13 of these patients were managed primarily with intervention. Amongst the subgroup of patients presenting in acute shock, the 30-day all-cause mortality was significantly higher in the group who underwent primary intervention (9 vs. 1, *p* = 0.006).

A total of 83 patients (32.4%) were in acute shock (shock index ≥ 1); 42 (51%) of these were managed conservatively and 41 (49%) were managed with intervention. Patients in acute shock had lower mean age, lower haemoglobin, lower platelet count, higher number of packed red blood cells, longer hospital length-of-stay and higher 30-day all-cause mortality (Table 3). Subgroup analysis of patients in acute shock demonstrated lower successful primary management with conservative management, however, this was not significant (88% vs. 95%, *p* = 0.26). Shocked patients managed conservatively were significantly more likely to be taking antiplatelet medication (14% vs 0%, *p* = 0.010), had higher mean platelet count (242 vs 148 g/L, *p* = 0.002), and had lower volume of mean packed red blood cell transfusions (4.8 vs 8.5, *p* = 0.020) (Table 3).

**Table 3:** Subgroup analysis of patients with a shock index > 1 (Acute Shock)

	Total	Conservative Primary Management	Interventional Primary Management	*P*-Value
Patients (number, percentage)	83	42 (51%)	41 (49%)	
Age in years (mean, SD)	54.4 (21.3)	58.0 (19.8)	50.8 (22.4)	0.12
*Sex (number, percentage)*				
Male	56 (67%)	28 (67%)	28 (68%)	0.88
Female	27 (33%)	14 (33%)	13 (32%)	
*Aetiology of haemorrhage (number, percentage)*				
Fall	17 (20%)	6 (14%)	11 (27%)	
Motor Vehicle Accident (MVA)	39 (47%)	23 (55%)	16 (39%)	
Sports	0 (0%)	0 (0%)	0 (0%)	
Other	25 (30%)	13 (31%)	12 (29%)	
Spontaneous:	2 (3%)	0 (0%)	2 (5%)	
*Location:* *(number, percentage)*				
Gluteal	29 (35%)	14 (33%)	15 (37%)	N/A
Other lower limb	20 (24%)	10 (24%)	10 (24%)	
Pectoral	6 (7%)	5 (12%)	1 (2%)	
Other upper limb	8 (10%)	2 (5%)	6 (15%)	
Breast	5 (6%)	4 (10%)	1 (2%)	
Superficial chest/abdominal wall	15 (18%)	7 (17%)	8 (20%)	
	17 (20%)	7 (17%)	10 (24%)	0.39
Antiplatelet therapy (number, percentage)	6 (7%)	6 (14%)	0 (0%)	0.01
Haemoglobin at presentation in g/L (mean, SD)	110 (25.0)	112 (24.7)	108 (25.4)	0.43
Haemoglobin at discharge in g/L (mean, SD)	105 (14.2)	107 (12.8)	102 (15.5)	0.15
Lowest systolic blood pressure (SBP) in mmHg (mean, SD)	82 (18.4)	82 (17.8)	81 (19.3)	0.84
Highest heart rate (HR) in beats per minute (mean, SD)	113 (24.4)	109 (21.0)	116 (27.1)	0.15
INR (mean, SD)	1.53 (0.7)	1.49 (0.7)	1.57 (0.7)	0.59
Platelet count in g/L (mean, SD)	194 (135.0)	242 (167.7)	148 (70.0)	0.002
Renal function (eGFR) in mL/min/1.73m2 (mean, SD)	72 (20.6)	71 (19.1)	73 (22.3)	0.64
Shock index (mean, SD)	1.41 (0.5)	1.36 (0.4)	1.46 (0.5)	0.28
Packed red blood cells (pRBCs) received (mean, SD)	6.7 (6.9)	4.8 (3.9)	8.5 (8.6)	0.02
Hospital length of stay (mean days, SD)	18.2 (16.5)	22.1 (20.2)	14.2 (10.4)	0.03
Successful primary management (number, percentage)	76 (92%)	37 (88%)	39 (95%)	0.26
30-day all-cause mortality (number, percentage)	10 (12%)	1 (2%)	9 (22%)	0.006

*SD* Standard Deviation. *eGFR* estimated glomerular filtration rate. *INR* International Normalized Ratio. *Shock Index* heart rate (HR) divided by systolic blood pressure (SBP)

## Discussion

This study showed that arterial bleeding in anatomically compressible sites can be successfully treated with conservative measures including manual compression and ice, evidenced by the 92.4% success rate of primary conservative management, comparable to the 91.8% success rate with intervention. Although patients that were managed with intervention were generally more clinically unwell, and subgroup analysis of patients in acute shock demonstrated a lower success rate of conservative management compared to intervention (88% vs. 92%), this was not statistically significant. Therefore, even for patients presenting in acute shock, conservative management is comparable to intervention. It should be noted that our data set comes from a large trauma centre, with an experienced trauma team comfortable in managing shocked patients with resuscitation measures as long as the site of bleeding can be promptly managed, including compression for bleeding at compressible sites.

30-day all-cause mortality was 6% of our patient cohort, with higher mortality in patients managed with intervention compared to those managed conservatively. However, patients managed with intervention were more likely to have other serious concurrent injuries, with the higher mortality likely reflective of these other injuries [16]. The deaths of the two patients in the conservatively managed group were related to their background comorbidities, and a change in the management of their bleeding sites would have unlikely changed the outcome. Furthermore, the mean age of our cohort was 60 years, and previous studies have demonstrated increased mortality in older patients, even with low level trauma, due to comorbidities and lower physiological reserves [17].

Limited previous data exists comparing conservative and invasive management in this patient cohort. Much of the current evidence stems from studies of military battlefield trauma, where the use of devices such as tourniquets to manage haemorrhage demonstrated positive outcomes [18, 19].

There are several considerations when deciding to treat a potentially compressible site with conservative measures. The volume of patients with small site arterial bleeding is relatively high, and many patients present to hospital outside of normal working hours [20]. Activation of an on-call interventional radiology or surgical team incurs cost and resources, which contributes to strain on busy hospital networks. In our study, the length of hospital stay in the conservatively managed group was shorter (11.8 vs. 12.5 days), although not significantly. In addition, frequent afterhours work has been shown to be a contributor to burnout [21]. It is also important to consider the risks of intervention, which include iatrogenic injury, exacerbation of bleeding, infection, and a visible wound with surgery, and contrast-induced acute kidney injury, dissection, thrombosis, and access site complications with embolisation. While these risks are small, they do materialise [22]. As with all clinical decisions, it is a balance between benefits and risks as well as informed consent. Importantly, as shown in this study, a conservative pathway is often viable.

Interventional radiologists (IRs) must assume clinical responsibility for patients from the moment of referral through to completion of treatment [23], which embodies the anecdote of “treat the patient, not the scan”. To make a blanket decision to embolise all patients with arterial bleeding is akin to avoiding clinical decision making and demoting IR to a technician role.

Our study has demonstrated the high success rate of conservative management in this patient cohort, which should give IRs confidence in their decision making when recommending conservative management as a primary management.

This study has limitations. Firstly, this was a retrospective non-randomised study, relying on historical records with limited understanding of clinical decision-making. There was heterogeneity in cohorts; for example, the primary intervention group were younger and haemodynamically more unstable, which likely influenced decision-making. Furthermore, concurrent injuries including displaced long bone fractures likely influenced decisions to proceed to surgical intervention, with haematoma treated concurrently. The higher mortality in the intervention cohort also likely reflected rates of associated injuries, rather than a direct outcome of intervention.

## Conclusion

Conservative management of arterial bleeding in anatomically compressible sites has a high success rate. Mechanical compression, application of ice, anticoagulation reversal and fluid resuscitation, are all treatments that can be immediately applied in the emergency setting, utilising the body’s inert anticoagulation properties to control the bleeding and prevent potentially unnecessary further procedures, even in acutely shocked patients. We encourage IRs to use their clinical decision-making skills when assessing patients with compressible haematomas and consider conservative measures, at least as a primary management.
